# Beyond the Bench: Building Blocks of Learning

**Published:** 2005-01

**Authors:** Amy Fitzgerald

Playing with blocks has long been a favorite pastime of children and one that parents often encourage as a means of developing reasoning, spatial acuity, and other skills. A program developed by the Community Outreach and Education Program (COEP) of the Massachusetts Institute of Technology (MIT) Center for Environmental Health Studies turns this childhood pastime into an effective method for teaching students about DNA and cellular processes by building models out of LEGO blocks.

Now a commercial product, the LEGO Life Science kits contain different-colored blocks representing the basic structural elements of DNA. So far, the kit series includes models of DNA, chromosomes, and photosynthesis. The kits were developed by Lexington public school teacher Kathleen Vandiver to bring the form and function of the double helix alive for middle-school students. Vandiver later joined the staff of the MIT COEP and has worked with the program to design a learning activity for students based on the kits called “The Shape of Life: From Helix to Chromosome.”

The activity begins with students identifying LEGOs that represent molecules of sugar, phosphates, and nucleotide bases. Using these pieces, they construct their own twisting model of the DNA ladder, with careful attention to base pairing. A brief overview of DNA replication follows, using the LEGO DNA structure as a simulation aid. Students then have individual exploration time to answer questions and investigate variations of their DNA model. Teachers may also add a mutation lesson.

Next, the students learn how DNA’s complex sequence is replicated prior to mitosis, and the lesson scales up to the LEGO Chromosomes kit to model the process of mitosis. The activity concludes with the study of structural components of chromosomes, including a discussion of genes and traits. With the Chromosomes kit, students can build a LEGO fish as a model to demonstrate how genes can be expressed in a living creature. The fish has only 3 chromosome pairs, rather than a human’s 23 pairs, so it’s easier to understand the relationship between genes and traits. Chromosomes, cell membranes, and spindle fibers are modeled in LEGOs as the students move through the stages of interphase, prophase, metaphase, anaphase, and telophase.

The kits can be used to teach many levels of students. Although originally designed for middle schoolers, they can be reassembled into a more advanced version for use at the college level. Several introductory biology classes at MIT have used the sets. Says Vandiver, “It is important to realize that many people need to be taught the basics in order to understand the issues in environmental health.” And what could be more basic—or fun—than playing with blocks?

The LEGO Life Science kits are available for purchase at **http://www.legoeducationstore.com/**.

## Figures and Tables

**Figure:**
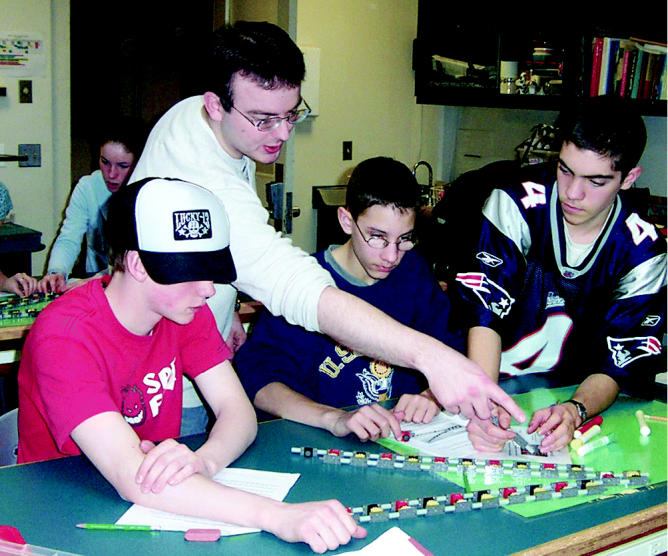
**Constructive thinking.** COEP researcher Luke Higgins (second from left) helps students build a DNA double helix from LEGOs.

